# Classification between normal and tumor tissues based on the pair-wise gene expression ratio

**DOI:** 10.1186/1471-2407-4-72

**Published:** 2004-10-07

**Authors:** YeeLeng Yap, XueWu Zhang, MT Ling, XiangHong Wang, YC Wong, Antoine Danchin

**Affiliations:** 1 HKU-Pasteur Research Centre, Dexter H.C. Man Building, 8 Sassoon Road Pokfulam, HongKong, China; 2 Cancer Biology Laboratory, Department of Anatomy, Faculty of Medicine. The University of HongKong, China; 3 Central Laboratory of the Institute of Molecular Technology for Drug Discovery and Sythesis, The University of HongKong, China; 4 Institute Pasteur, Unité de Génétique des Génomes Bactériens, 28 rue du Docteur Roux, 75724 Paris Cedex 15, France

**Keywords:** Hepsin, Adipsin, Monocyte-derived neutrophil-activating protein, Prostate cancer, Colon cancer

## Abstract

**Background:**

Precise classification of cancer types is critically important for early cancer diagnosis and treatment. Numerous efforts have been made to use gene expression profiles to improve precision of tumor classification. However, reliable cancer-related signals are generally lacking.

**Method:**

Using recent datasets on colon and prostate cancer, a data transformation procedure from single gene expression to pair-wise gene expression ratio is proposed. Making use of the internal consistency of each expression profiling dataset this transformation improves the signal to noise ratio of the dataset and uncovers new relevant cancer-related signals (features). The efficiency in using the transformed dataset to perform normal/tumor classification was investigated using feature partitioning with informative features (gene annotation) as discriminating axes (single gene expression or pair-wise gene expression ratio). Classification results were compared to the original datasets for up to 10-feature model classifiers.

**Results:**

82 and 262 genes that have high correlation to tissue phenotype were selected from the colon and prostate datasets respectively. Remarkably, data transformation of the highly noisy expression data successfully led to lower the coefficient of variation (CV) for the within-class samples as well as improved the correlation with tissue phenotypes. The transformed dataset exhibited lower CV when compared to that of single gene expression. In the colon cancer set, the minimum CV decreased from 45.3% to 16.5%. In prostate cancer, comparable CV was achieved with and without transformation. This improvement in CV, coupled with the improved correlation between the pair-wise gene expression ratio and tissue phenotypes, yielded higher classification efficiency, especially with the colon dataset – from 87.1% to 93.5%. Over 90% of the top ten discriminating axes in both datasets showed significant improvement after data transformation. The high classification efficiency achieved suggested that there exist some cancer-related signals in the form of pair-wise gene expression ratio.

**Conclusion:**

The results from this study indicated that: 1) in the case when the pair-wise expression ratio transformation achieves lower CV and higher correlation to tissue phenotypes, a better classification of tissue type will follow. 2) the comparable classification accuracy achieved after data transformation suggested that pair-wise gene expression ratio between some pairs of genes can identify reliable markers for cancer.

## Background

Tumor development is a process in which gene expression is modified, causing abnormal cell behaviour [1]. Many techniques have been developed to identify abnormalities of gene expression, as reflected by abundance of *mRNA *transcripts between normal and tumor. The completion of the Human Genome Project and advances in DNA-array technology have allowed highly parallel genetic analyses to take place on a genome-wide scale. They have revolutionized the way tumors are studied, and promised to provide a better and more thorough understanding of the underlying mechanisms for tumorigenesis. Eventually, they will lead to more comprehensive diagnosis/prognosis of tumor with more effective therapeutic interventions.

Despite its advantages, the DNA-array technology poses three major challenges that render the interpretation of expression data less efficient than expected. Firstly, the gene expression data is inherently variable due to various factors that either depend on biological factors that remain difficult to control (cross-contaminated samples of tumor and normal cells), or depend on difficulties in setting up of the experiment (RNA extraction) [2]. These drawbacks interfere with the subsequent array analysis aimed to identify reliable markers that best correlate with the tissue phenotypes. Efforts have been devoted to address these drawbacks by incorporating various raw data scaling, data filtering, normalization and improvement of the classifier algorithm [3]. Promising results have been reported claiming near-perfect classification accuracy [4]. However, the usually small number of samples per class in most studies and the highly biased cross validation procedures cast doubt on the classification accuracy in terms of their statistical significance [5]. This statistical constraint creates a further challenge for DNA-array technology where the number of features in arrays is in thousands while tissue samples are available in limited number. This causes high probability for any classification to be correct by chance alone. Thirdly, although it has been recently established that genes segregate into clusters of interacting networks [6] instead of acting as one single entity, most cancer DNA-array studies have only investigated single gene aberration (up/down-regulated) when comparing tumor expression profiles to their corresponding normal tissue controls. In an interesting study, B∅ and Jonassen tried to circumvent some of these difficulties by investigating genes in pairs. They demonstrated that gene pairs can be used to improve discrimination between different tissue classes [7]. This idea of studying genes in pairs, or even in higher order clusters, should be explored further to reveal new features of complex expression profiling datasets.

In this study, we introduced a novel data transformation meant to investigate relationships between pair-wise gene expression ratios and tissue phenotype within a given experiment. With this procedure, we aimed to discover strong cancer-related signals (features) that exist in the form of pair-wise ratios (or higher order relationship when we extend to N-feature model classifier for N>2) in a given sample, while improving the signal to noise ratio of the dataset by minimizing its coefficient of variation (CV). The underlying concept for adopting pair-wise gene expression ratios as the discriminating axes for tissue type classification is that an experiment is self-consistent (in terms of factors affected either by the biology of the phenomenon of interest, or of the experimental setting, or both). With this approach we could "subtract" correlated variations by considering the sample as a whole, without making inferences such as those needed for normalization. Basically, we avoided studying gene expression in an absolute term because this requires robust normalization method to account for arrays from different experiments, different platforms and different profiling technologies. By resorting to analyze features in the form of ratios, we attempted to minimize the effect of normalization and look for co-varying signals in each experiment.

## Methods

### Colon and prostate cancer datasets

The 62 colon cancer sample dataset is composed of measurements for 1,988 gene probes, of which 40 were labelled as tumor and 22 were labelled as normal. The samples were collected from patients, their RNAs were extracted and hybridised to Affymetrix Hum6000 arrays. Please refer to paper [8]. The normalized dataset can be downloaded at .

The 102 prostate cancer sample dataset is composed of measurements for 12,600 gene probes, of which 52 were labelled as tumor and 50 were labelled as normal. The samples were collected from patients, their RNAs were extracted and hybridised to Affymetrix U95Av2 arrays. Please refer to paper [9]. The normalised dataset can be downloaded at .

Both datasets were pre-processed to eliminate those probe pairs that showed significant fluctuation in their hybridisation signals (those greater than 3 standard deviation away from the mean for their ESTs, and the probes pairs that showed an overall higher intensity in their mismatch probe cells (MM) than their corresponding perfect match probe cells (PM); these probe pairs indicate non-specific hybridisation by background RNAs). Both datasets used average intensity as quantitative measurements of the level of gene expression. Base-10 logarithmic transformations were performed for each dataset.

### Initial gene selection

For downstream classification analysis, we extracted only the genes whose expression pattern correlated strongly to the tissue phenotype. To achieve this, we first calculated the correlation coefficient *r*_i _(Equation 1) for each gene *i *using the full dataset, and ranked the genes according to their correlation coefficient *r*_i_. For the calculation of *r*, we assigned a number to each tissue phenotype: 1 for normal tissue and 10 for cancer tissue. After obtaining the correlation coefficients for all genes, we used a simple threshold value (|*r*|>0.4) to select the set of cancer-related genes. There were two reasons for set the threshold value at 0.4. When lower thresholds were used, we incorporated many genes that were not known to be cancer-related (data not shown). Furthermore, too many genes will later cause computer tractability problem when we calculate their pair-wise gene expression ratio for each tissue sample and later the N-feature model classifier. At |*r*|>0.4, we were able to account for most of previously known cancer related genes.


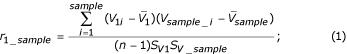


where *V*_1 _is a vector representing the gene expression pattern for gene #1; *V_sample _*is the dichotomous representation of tissues; *S_V1_*and *S_sample _*standard deviation of *V_1_*, *V_sample_*; 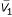, 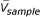 are the mean of *V_1_*, *V_sample_*.

### Transforming the gene expression data to investigate the expression equilibrium between genes pairs

**Figure 1:**
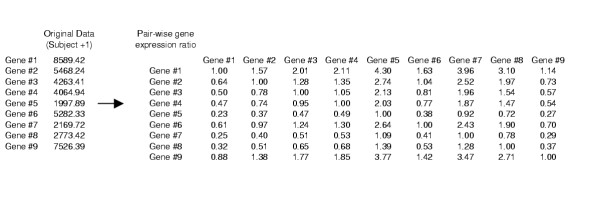
Transformation of gene expression data.

The raw expression data within a sample tissue was transformed into measurement of the pair-wise gene expression ratio for any combinatorial pairs of genes. For the 1,988 gene expression intensities for each sample (*e_1_*, *e_2_*...*e_1988_*), there are ^1988^C_2 _combinations (*e_1_*/*e_2_*, *e_1_*/*e_3_*...) of pair-wise gene expression ratios (Figure 1). This transformed matrix is referred to as *M*. Each row/column corresponds to a specific gene and the entry at the intersection of row X and column Y corresponds to the expression equilibrium between gene X and gene Y. Such matrix has a diagonal entry of value 1 because e_1_/e_1 _equals to unity.

### Feature partitioning method [4] for classification of normal/tumor tissues using single gene expression

**Figure 2:**
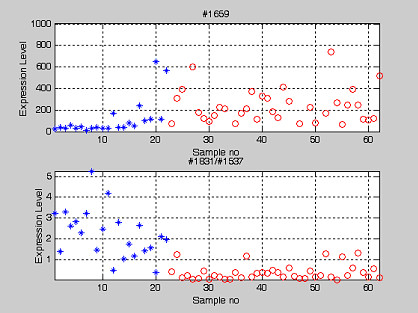
**Potential colon cancer gene markers: The expression of single gene and the transformed pair-wise gene expression ratio. **Potential gene marker for colon cancer tissue (#1659-Human monocyte-derived neutrophil-activating protein (MONAP) *mRNA*). However, we observed that the pair-wise gene expression ratio (#1537/#1831- ratio between vascular endothelial growth factor and gelsolin precursor) has better discriminating efficiency as tabulated in Table 7. ('*' and 'o' represent normal and cancer tissue type respectively).

Regarding the Feature Partitioning Method (FPM), in order to discriminate between the normal/tumor tissues based on specific feature *i *(single gene expression), the first step is to determine the threshold value, *T_i_*, that can optimally splits all the tissue samples into tumor and normal tissue. The FPM algorithm has a recursive version [4], in which a decision tree depicting the classification rules for tissue samples was generated recursively. Both methods differ in the way *T_i_*s are derived. Nonetheless, they are very intuitive and non-parametric in nature. Also, they restrict no priori distribution patterns for features used. We adopted the simple FPM for tissue classification where each feature was treated individually. There are two criteria for deriving a valid threshold value *T_i _*for each feature. First, it has to delineate correctly (discriminating efficiency = 100%) the one-dimensional region (R_feature_i_) for either all the normal/tumor tissues using all tissue samples. Secondly, it has to minimize the percentage of false prediction for the other tissue type. Take gene #1659 for example. To fulfill the two aforementioned criteria, it was determined that the region greater than 63.7 (R_#1659_) incorporates all the tumor samples (Figure 2). It classified correctly all tumors (discriminating efficiency = 100%) with an overall false prediction of 13.9% in the normal set. This was performed repeatedly for all features until all the threshold values (*T*_*i...all *features_) were determined.

Now, to classify an unknown sample using 2-feature model classifier, a combination of any two features and their corresponding pre-determined threshold values *T_i_*s (selected from *T*_*i...all *features _for each dataset) were recruited. The outcome of the tissue class will be determined depending on whether one/both the expression values of the unknown sample fall completely in either the normal/cancer region (R_feature_i_). This is to say that if any of the two features from the unknown sample meets the criteria (R_feature_i_) to be either normal/tumor tissue type (based on our definition, R_feature_i _is a region with 100% discriminating efficiency for a specific tissue type), the unknown sample will be assigned to be normal/tumor respectively. This is repeated exhaustively for all possible combinations constituting of any two features. The procedure will be repeated for all tissue samples to evaluate the overall classification accuracy for 2-feature model classifier. In total, we evaluated the classification of tissue samples based on different combinations of N genes and investigated the classifiers up to 10-feature model classifier.

### Classification of normal/tumor tissues using transformed datasets

The classification procedures and the two criteria for determining the threshold value were the same as explained in previous paragraph. The only difference here is that the definition of "feature" refers to pair-wise gene expression ratio derived from lower/upper triangular matrix of *M*. Take the ratio #1537/#1831 for example. To fulfill the two aforementioned criteria, it was determined that the region greater than 0.755 (R_#1537/#1831_) incorporates all the tumor tissue samples (Figure 2). It classifies correctly all tumor tissue samples with a false prediction of 6.4%. This is performed repeatedly for all entries in *M *until all the threshold values are determined.

Now, to classify an unknown sample using 2-feature model classifier, a combination of any two features (pair-wise gene expression ratio) and their corresponding pre-determined threshold values *T_i_*s (selected from *T*_*i...all *features _for each dataset) were recruited. The outcome of the tissue class will be determined depending on whether one/both the expression values of the unknown sample fall completely in either the normal or cancer region (R_feature_i_). This is to say that if any of the two features (pair-wise gene expression ratio) from the unknown sample meets the criteria (R_feature_i_) to be either normal/tumor (based on our definition, R_feature_i _is a region with 100% discriminating efficiency for a specific tissue type), the unknown sample will be assigned to be normal/tumor respectively. This is repeated exhaustively for all possible combinations constituting of two features. The procedure will be repeated for all tissue samples to evaluate the overall classification accuracy for 2-feature model classifier. In total, we evaluated the classification of tissue samples based on different combinations of N genes and investigated the classifiers up to 10-feature model classifier.

### Constructing the relationship tree for the top 25 genes

We calculated the cross correlation coefficient *r *(Equation 1) for all pair combinations of the top 25 genes listed in Table 6 and Table 7. Prior to the construction of a relationship tree for the top 25 genes for colon and prostate cancer, the cross-correlation coefficient was used to construct the pair-wise distance matrix *D*. Each entry in the pair-wise distance matrix was measured by the value of (1-*r*). Each row/column corresponds to a specific gene and an entry at the intersection of row X and column Y corresponds to the distance of gene expression between gene #X and gene #Y. Such matrix has a diagonal entry of value 0. Only the lower/upper triangular matrix of *D *is required to construct the relationship tree. After obtaining lower/upper triangular matrix of *D*, the neighbor-joining method (NJ) algorithm was used to construct the relationship tree [10].

### Computer hardware and software

A Sun Fire 6800 Server  with 24 CPUs (each running with a clock speed of 900 MHz) was employed throughout this study. The computation of correlation coefficient and classification procedures were implemented using the Matlab Technical Programming language (Matlab programs can be downloaded at .

## Results

After initial gene selection, respectively 82 and 262 genes (|*r*|*>*0.4) were selected from the colon and prostate dataset for downstream analysis (Table 1 and Table 2). Topping the list in both tables were genes that have been found to be either over-expressed/under-expressed in tumors [11]. The first three genes most correlated to cancer in the colon dataset were heavy chain of non-muscle myosin, human monocyte-derived neutrophil-activating protein (MONAP) and human desmin genes. This agrees with the findings from [12,13] that used other statistical tests (*z*-score, *t*-test) in a comparable analysis. The heavy chain of non-muscle myosin, denoted as the embryonic smooth muscle myosin heavy chain (SMemb), was found to be down-regulated in cancer. It was also determined experimentally to be a target for the protein encoded by the metastasis-related mts-1 gene [14]. Furthermore, it was demonstrated recently by 5'RACE analysis that heavy chain of non-muscle myosin interacts with ALK genes that have tyrosine kinase activity and oncogenic properties [15]. The human monocyte-derived neutrophil-activating protein (MONAP, interleukin-8), was second on the list. It was significantly up-regulated in the tumor compared to the normal samples. This protein has been linked to the progression of several human cancer types [16]. It was believed that over-expression of MONAP plays an important role in tumor angiogenesis and tumor aggression. The human desmin gene is the third on the list, and it was found to be down-regulated in tumor. Interestingly, this gene also showed significantly reduced expression in other cancer types such as the melanoma cell line [17].

From the prostate dataset, the most cancer-correlated gene is the human hepatoma gene coding for serine protease hepsin. Brief literature search in PubMed showed that hepsin is a well-characterized transmembrane protease that is expressed at high level in tumor. Three separate studies identified hepsin as a significant cancer biomarker that can be used for cancer diagnosis [18]. The second gene on the list was the human mitochondrial matrix protein P1. This gene has been correlated to different cancer types with consistent up-regulation in tumor [13]. The third gene is the carcinoma-associated antigen GA733-2, which was among the 216 cancer markers identified by Ernst's group in Germany [19].

### Effect of data transformation on coefficient of variation

To date, reliable markers with low coefficient of variation (CV) are generally lacking. Discovering robust cancer marker is crucial for the purpose of successful cancer diagnosis. We investigated the CV between samples after data transformation: the lowest CVs decreased to 16.5% in the colon dataset while it increased to 25.8% for the prostate dataset (Table 3 and Table 4). Topping the list for both dataset were the pair-wise gene expression ratio for genes #119/#54 (elongation factor 1-delta and 40S ribosomal protein S24) and #10614/#5871 (zq58b03.r1 *Homo sapiens cDNA *and nuclear matrix protein NXP2), which revealed informative pair-wise gene interaction in relation with their corresponding tissue phenotypes. They reflected how cell adjusts to their pair-wise product in response to physiological changes. Based on these observations, we found that the relative abundance between the numerator and denominator exhibited a strong mutual dependency, and had strong correlation to tissue phenotype. For pair-wise gene expression ratio #119/#54, the elongation factor 1-delta is involved in a sequence of events during the decoding of *mRNA *on the ribosome [20]. For the ratio of #10614/#5871, it corresponds to novel genes that do not yet have known function. A search in the DNA non-redundant (nr) database for gene #10614 yielded 83% DNA identity to a segment on chromosome 9. On the other hand, a search in non-redundant (nr) database for #5871 revealed 72.3% DNA identity to the *cDNA *of mouse that incorporates proteins involved in chromosome partitioning and cell decision [21].

Prior to data transformation the lowest coefficients of variations for single gene expression were 45.3% and 24.5% for colon and prostate datasets respectively. When using the data transformation we proposed, significant improvement was achieved in the colon dataset. Interestingly, this was followed by an improved data correlation to the tissue phenotype as well as to the classification efficiency. We did not observe a similar improvement of the CV, data correlation to tissue classes or classification efficiency in the prostate dataset.

### Correlations of the single gene expression and pair-wise gene expression ratio

**Figure 3:**
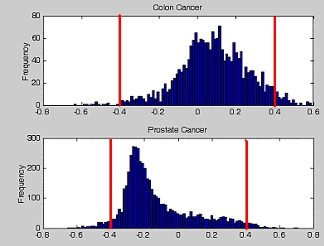
**The histogram for correlation of coefficient *r *between single gene expression and the tissue types for the colon and prostate tissue cancer. **The distribution shows coefficient of correlation between single gene expression and cancer phenotype. Their extrema of correlation coefficient |*r*|>0.4 (represented in red lines) were extracted for downstream data analysis.

**Figure 4:**
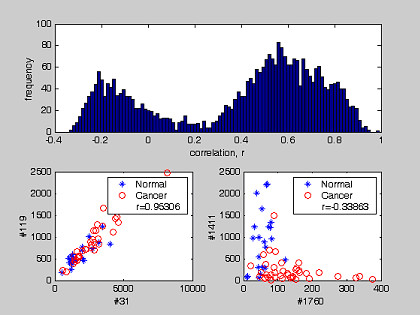
**The distribution of cross-correlation between two single gene expression patterns in colon dataset. **The distribution shows the coefficient of correlation between expression patterns for any pair of gene markers. Their extrema scenarios were also plotted with their corresponding *r *value.

**Figure 5:**
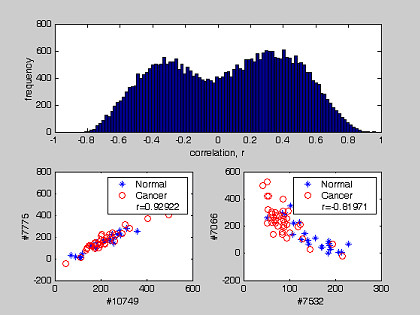
**The distribution of cross-correlation between two genes expression patterns in prostate dataset. **The distribution shows coefficient of correlation between any pair of gene markers. Their extrema plots of correlation coefficient were also plotted with corresponding *r *value.

The distribution of correlation coefficients between genes and tissue phenotypes for the colon and prostate datasets is shown in Figure 3. The distributions are positively and negatively skewed for both datasets. The two red lines separate genes with |*r*| >0.4 from the bulk (Table 1 and 2). They retained respectively 82 and 262 genes from the colon and prostate datasets. To study the possible interaction between pair-wise genes, we estimated the statistical correlation of gene expressions. Both the distributions for the correlation coefficient and the extreme cases are shown in Figures 4 and 5. Both figures emphasize the true nature of gene-gene co-regulations – a complex biological mechanism, that most often has been over-simplified when we treat the gene expression as an independent variables [22]. For example, Figure 4 and Figure 5 suggested that the expressions of genes belonging to a common subset are most likely correlated to each other (e.g.: Gene #31 *vs *#119 in colon cancer (*r *= 0.95306) and gene #7775 *vs *#10749 in prostate cancer (*r *= 0.92922)). It should be pointed out that the two humps in the probability density function are not zero-centered, but concentrated at non-zero correlation *r*. For colon dataset, positive correlation was the dominant type. For prostate dataset, a balanced distribution in their gene correlation was observed. We determined that some improvement in tissue classification is achieved when pair-wise gene expression ratio was used as discriminating axes instead of using a single gene expression (Figure 2). The reason is that pair-wise gene expression ratio has higher correlation to tissue phenotype with lower CV (Table 5).

### Gene expression and tissue type correlation

Several previous studies have already endeavored to identify correlations between specific gene expression and cancerous transformation [4,13,23]. In the present study, we identified several novel target genes that clearly distinguish the two different tissue phenotypes with high discriminating efficiency (>74%) (Table 6 and Table 8). Some of those have previously been documented in studies that did not involve expression profiling as cancer related genes (Human monocyte-derived neutrophil-activating protein (MONAP) and Human hepatoma *mRNA *for serine protease hepsin), others (Human gene for heterogeneous nuclear ribonucleoprotein (hnRNP), P24480 CALGIZZARIN, Human mitochondrial matrix protein P1, Human *mRNA *for aldose reductase and human adipsin) have not been identified from *in-silico *studies of tissue DNA-array expression data. The cancer related genes for colon and prostate cancer were ranked according to their discriminating predictive power. The list should provide hints for researchers during selection of molecular target for diagnostic, prognostic or attempts to cure the disease. Overall classification results and accuracies for each N-feature model classifier across two datasets were reported in Table 6, 7 and 8. In the following section, we will discuss a few important genes or pair-wise gene expression ratios from Table 6 and Table 7 that resulted in the optimum classification accuracy (Table 8B). They are the most efficient combination of discriminating axes for classifying tissue types because they delineate correctly all the normal/tumor tissues with the lowest percentage of false prediction.

For the sake of brevity, we will discuss three single gene expressions and two pair-wise gene expression ratios from colon cancer. For prostate cancer, two single gene expressions and two pair-wise gene expression ratios will be discussed.

For colon cancer single gene expression, three axes for discriminating tissue types are: 1) Human monocyte-derived neutrophil-activating protein (MONAP); 2) Human desmin gene and 3) Human cysteine-rich protein (CRP) gene. Their threshold values were determined to be 62.73, 2787.0 and 749.4 respectively.

For colon cancer pair-wise gene expression ratio, the two axes for discriminating tissue types are: 1) #1831/#1537 and 2) #753/#768. Their threshold values were reported to be 1.32 and 1.85 respectively.

For prostate cancer individual gene expression, the two axes for discriminating tissue types are: 1) Human hepatoma mRNA for serine protease hepsin and 2) Human adipsin. Their threshold values were reported to be 115.0 and 182.0 respectively.

For prostate cancer pair-wise gene expression ratio, the two axes for discriminating tissue types are: 1) #6185/#5840 and 2) #6185/#6749. Their threshold values were reported to be 2.69 and 2.55 respectively.

**Figure 6:**
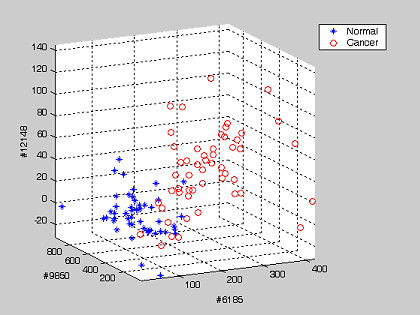
**Prostate dataset: an example showing the projection of 102 tissue samples on the top three discriminating axes of the single gene expression patterns. **The gene numbers are shown as the axis labels. The threshold values *T_i _*for normal tissues on each axis are tabulated on Table 7.

**Figure 7:**
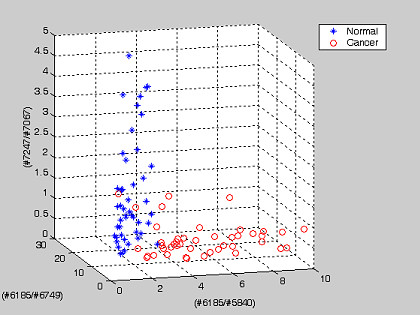
**Prostate dataset: an example showing the projection of 102 tissue samples on the top three discriminating axes of the pair-wise gene expression ratio. **The gene numbers are shown as the axis labels. The threshold values *T_i _*for normal tissues on each axis are tabulated on Table 7.

To illustrate graphically the result of tissue classification, two examples, each based on three genes or pair-wise gene expression ratios that altogether yielded the optimum classification efficiency for the prostate cancer are shown (Figure 6, Figure 7).

### Constructing the relationship tree for top 25 gene for colon and prostate cancer

**Figure 8:**
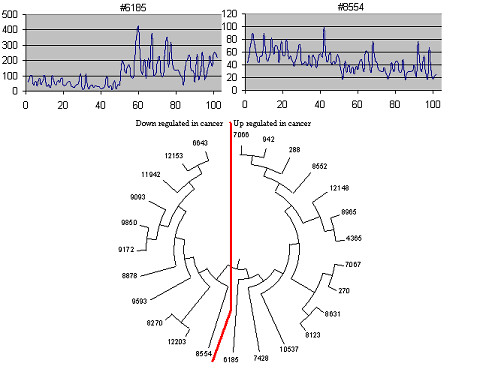
**Inter-relationship of gene expression gene expression for top 25 prostate cancer genes extracted from Table 8. **The tree structure was derived using neighbor-joining algorithm [10]. Two clusters of gene expression were observed, namely the up-regulated (#6185) and down – regulated (#8554) genes in cancer tissues.

The relationship tree for top 25 genes listed in Table 6 and Table 7 were constructed based on the cross-correlation between gene expressions (Figure 8). We employed the established 'neighbor-joining' clustering method [10] to group different genes based on their correlated expression patterns across all tissue samples (meaning that genes expression that are correlated will appear in the same branch of the clustering tree), using a novel distance measurement to quantify how change in the expression for one gene interfered with that of another gene. The principle of this method is to cluster pairs of operational taxonomic units (OTUs [=neighbors of similar gene expression]) that minimize the total branch length at each stage of clustering of OTUs starting with a star-like tree. Figure 8 revealed two major clusters of genes. The first cluster corresponded to down-regulated genes, the second cluster represented up-regulated genes. Also, the most efficient discriminating axes (feature genes) reside at the basal position for each cluster. In bacteria many genes are co-expressed as single transcription units. This was used as a control study to validate the methodology of grouping genes, we implemented this distance measurement on bacteria gene arrays (*B. subtilis *and *E. coli*) and successfully determined the co-regulated operon gene structures (supplementary file #1).

## Discussion

### Data transformation to investigate pair-wise gene expression ratios

As the expression profiling technologies mature, the identification of significant cancer-related signals from noisy datasets (characterized by a high CV) remains a major challenge. In particular, a robust normalization method is critical to ascertain that arrays from two experiments are comparable with minimum noise prior downstream analysis. However, the existing normalization methods pose limitations due to the lack of good models to account for sources of experimental and biological variations [24]. Hoffmann et al. [25] employed different normalization methods to analyse the same dataset, and demonstrated that the numbers of genes detected as differentially expressed differed by a huge factor depending on which normalization methods used. The problem is exacerbated further by the presence of different array formats, experimental designs and methods.

Here, instead of resolving to single gene expression, that depends heavily on normalization, for tissue classification, we presented a transformation method that uses pair-wise gene expression ratios within the same experiment as the discriminating axes. By doing so, we aimed to minimize the influence of different normalization methods considering that an experiment is self-consistent with the same factors affecting all genes in the same fashion. The rationale is that even when the normalization methods differ between two array experiments, their pair-wise gene expression ratios within the same experiment will remain relatively stable. If reliable cancer-related signal, exist in the form of pair-wise gene expression ratio, were indeed discovered successfully, they will be relatively independent from the normalization method used on a dataset.

The improvement in CV (Table 3) and overall classification accuracy (Table 7) for colon dataset after introduction of data transformation signifies two implications: First, the transformation is able to increase the signal to noise ratio (SNR) of the cancer related signal because the resulted pair-wise gene expression ratios correlate stronger to tissue phenotype. Second, because the pair-wise gene expression ratios are less dispersed than single gene expression, using the pair-wise gene expression ratios to classify tissue types will be much more reliable and accurate (Table 8). Despite the benefits mentioned, this data transformation introduced a computational limitation due to the enormous amount of feature combinations to be processed, especially when N-feature model classifiers for N>4 are considered (If 100 features are selected, and 10-feature model classifier is investigated, the search space will be ^100^C_10_= 1.731030945644000 × 10^13 ^different combination of features). As a result, more computation time will be required to search all possibilities. As an example, the discriminating axes that accounted for the optimum accuracy in 1 to 3-feature model classifier are reported in Table 9.

Regarding the high classification accuracy reported in Table 8, it should be stressed that this was achieved by involving all tissue samples during the derivation of the threshold value, *T_i_*, in the feature selection procedure. In other word, instead of adopting the more conservative classification accuracy test where only a subset of tissue samples are used to derive a set of classification criteria (threshold values), we adjusted our methodology to use all tissue samples so that our results are unbiased (when comparing the outcome from single gene and pair-wise gene ratio) and in-line with our objective that is to compare the classification efficiency between single gene and pair-wise gene ratio. Admittedly, we have a noisy dataset whereby selecting a subset of tissue samples that are a representable population for the entire dataset remains a challenge [5] (given that we have a small and unbalanced dataset, particularly the colon dataset). Eventually, we might run into ambiguous/contradicting results using a different population subset of tissue samples. Furthermore, we might miss important features (single gene expression/ pair-wise gene expression ratio) because of the biased training dataset. By including all tissue samples for both studies (single gene and pair-wise gene ratio), we aimed to derive the most reliable threshold values and classified tissue samples based on them. Since the same methodology was applied for both studies, the comparison of classification efficiency is valid and will reflect how well each feature (single gene and pair-wise gene ratio) can be used to delineate tissue samples.

### The implication derived from the classification results

For colon dataset, three axes for discriminating tissues are: 1) Human monocyte-derived neutrophil-activating protein (MONAP); 2) Human desmin gene and 3) Human cysteine-rich protein (CRP) gene. The association of the first two genes and cancer biology had been discussed earlier. We will discuss the Human cysteine-rich protein gene. The expression and induction of this protein has been associated with protection against DNA damage, oxidative stress and apoptosis [26]. In the colon dataset, we observed down-regulation of this protein in tumor. This suggested lack of protection against DNA damage.

For colon cancer pair-wise gene expression ratio, the two axes for discriminating tissues are: 1) #1831/#1537 and 2) #753/#768. Using these two axes, 98.4% of the tissue samples can be classified correctly. The expression ratio between #1831 (gelsolin precursor) and #1537 (vascular endothelial growth factor) was able to discriminate 93.6% of the total tissue data. The vascular endothelial growth factor was determined recently to be a plausible biomarker for colon cancer [27]. Gelsolin had been found to suppress tumorigenicity in different cancer samples, including lung, bladder and breast [28]. When they were used individually as a discriminating axis, they were only able to classify correctly 66.1% and 67.7% of all tissue samples. Furthermore, the expression ratio between #753 (Human cysteine-rich protein) and #768 (the macrophage migration inhibitory factor) was able to discriminate 90.3% of total tissue type. The human cysteine-rich protein was discussed in the previous section. The macrophage migration inhibitory factor (MIF) functions as a pluripotent cytokine involved in broad-spectrum pathophysiological events in association with inflammation and immune responses. Several reports, including ours, have suggested that MIF is also involved in tumorigenesis [29]. When they were used individually as single discriminating axis, they were only able to classify correctly 83.9% and 66.1% of all tissues.

For prostate cancer single gene expression, the two axes for discriminating tissues are: 1) Human hepatoma *mRNA *for serine protease hepsin, and 2) Human adipsin. The first gene was discussed in the previous paragraph. For the second gene, adipsin had also been suggested by Chow et al. [30] as a good cancer marker for studying the basic biology of cancer.

For prostate cancer pair-wise gene expression ratio, the two axes for discriminating tissues are: 1) #6185/#5840 and 2) #6185/#6749. Using these two axes, all tissue samples can be classified correctly. The expression ratio between #6185 (Human hepatoma *mRNA *for serine protease hepsin) and #5840 (*Homo sapiens mRNA *for KIAA1109 protein) was able to discriminate 92.2% of total tissues. The human hepatoma *mRNA *for serine protease hepsin had been determined to be an important marker for cancer cell development [11,18]. The KIAA1109 protein is an unknown protein in human chromosome four [31]. A homology search against the non-redundant databases yielded no significant hit to known genes. When they were used individually as a discriminating axis, they were only able to classify correctly 86.3% and 61.8% of all tissues. On the other hand, the expression ratio between #6185 (Human hepatoma *mRNA *for serine protease hepsin) and #6749 (*Homo sapiens mRNA *for KIAA1055 protein) was able to discriminate 90.10% of total tissues. The human hepatoma *mRNA *for serine protease hepsin was discussed in the previous section. The KIAA1055 protein is an unknown protein in human chromosome 15 [21,31]. A homology search against the non-redundant databases yielded 40.7% DNA identity to a novel human *cDNA *that had been found to function as a cancer inhibiting protein [21]. When they were used individually as a discriminating axis, they were only able to classify correctly 86.3% and 62.8% of all tissues.

## Conclusion

By comparing the tissue classification methods based on the single gene expression and the pair-wise gene expression ratio in two microarray datasets, we reached the following conclusions:

1. The minimum coefficient of variation decreased from 45.33% to 16.53% for colon dataset but increased marginally from 24.54% to 25.78% in prostate dataset.

2. The correlation coefficient, *r*, of the discriminating axis that correlates maximally to the tissue phenotype improves from 0.63 to 0.79 and 0.71 to 0.75 in colon and prostate dataset respectively.

3. The optimum accuracy for 1-feature model classifier (using single gene or pair-wise gene expression ratio as discriminating axis) improved from 87.1% to 93.55% in colon dataset. In prostate dataset, nine out of the top 10 discriminating axes showed significant improvement. The mean accuracy for 1-gene classifier improved from 76.8% to 91.2% and 75.8% to 81.9% in both datasets.

4. The comparable classification accuracy achieved after data transformation suggested that there exist some cancer-related signals in the form of pair-wise gene expression ratio, especially prominent in the colon dataset.

5. Through the single gene analysis, we identified key biomarkers that agree with the findings by other researchers. In addition, study on gene-to-gene correlation and the classification outcome based on the pair-wise gene expression ratio suggested that genetic network within a cluster of cancer-related genes should be explored further.

## Competing interests

The authors declare that they have no competing interests.

## Authors' contributions

YLY proposed the idea, participated in the design, performed the statistical analysis and wrote the first draft of the manuscript.

AD participated in the design and overall coordination of this study as well as in the writing of the manuscript.

XWZ participated in the design of the study.

YCW, XHW and MTL participated during the revision phase of this study.

All authors read and approved the final manuscript.

## Pre-publication history

The pre-publication history for this paper can be accessed here:



## Figures and Tables

**Table 1:** Colon cancer: the gene retained for classification of tissue types. This table contains the genes and their descriptions. The key genes are selected based on how correlated their average intensity to the normal and tumor tissues. The genes are placed in the order of descending correlation coefficient *r*. Ten key genes are reported, the complete table can be downloaded at . Entire data for the experiment can be downloaded from .

No. on array	Gene accession number with correlation >0.4 to cancer tissue type	Info	Correlation
481	R87126	MYOSIN HEAVY CHAIN, NONMUSCLE (Gallus gallus)	0.6327
1659	M26383	Human monocyte-derived neutrophil-activating protein (MONAP) mRNA, complete cds.	0.5853
241	M63391	Human desmin gene, complete cds.	0.5848
1760	H08393	COLLAGEN ALPHA 2(XI) CHAIN (Homo sapiens)	0.5760
1030	R36977	P03001 TRANSCRIPTION FACTOR IIIA ;.	0.5741
1411	J02854	MYOSIN REGULATORY LIGHT CHAIN 2, SMOOTH MUSCLE ISOFORM (HUMAN);contains element TAR1 repetitive element	0.5680
1759	J05032	Human aspartyl-tRNA synthetase alpha-2 subunit mRNA, complete cds.	0.5670
613	X12671	Human gene for heterogeneous nuclear ribonucleoprotein (hnRNP) core protein A1.	0.5583
365	Z50753	H.sapiens mRNA for GCAP-II/uroguanylin precursor.	0.5494
753	M76378	Human cysteine-rich protein (CRP) gene, exons 5 and 6.	0.5354

**Table 2:** Prostate cancer: the key gene retained for classification of tissue types. This table contains the genes and their descriptions. The key genes are selected based on how correlated their average intensity to the normal and tumor tissues. The genes are placed in the order of descending correlation coefficient *r*. Ten key features were shown, the complete table can be downloaded at . Entire data for the experiment can be downloaded from .

No. on array	Gene probe with correlation >0.4 to cancer tissue type	Info	Correlation
6185	37639_at	Cluster Incl. X07732:Human hepatoma mRNA for serine protease hepsin	0.7119
8965	37720_at	Cluster Incl. M22382:Human mitochondrial matrix protein P1 (nuclear encoded) mRNA, complete cds M93036 /FEATURE=mRNA /DEFINITION=HUMGA7A08	0.7018
12148	575_s_at	Human (clone 21726) carcinoma-associated antigen GA733-2 (GA733-2) mRNA	0.6917
6462	38634_at	Cluster Incl. M11433:Human cellular retinol-binding protein mRNA	0.6514
10138	41288_at	Cluster Incl. AL036744:DKFZp564I1663_r1 Homo sapiens cDNA	0.6367
12153	556_s_at	M96233 /FEATURE=expanded_cds/DEFINITION=HUMGSTM4A Human glutathione transferase class mu number 4 (GSTM4) gene	0.6217
6866	39756_g_at	Cluster Incl. Z93930:Human DNA sequence from clone 292E10 on chromosome 22q11-12. Contains the XBP1 gene for X-box binding protein 1 (TREB5), ESTs, STSs, GSSs and a putative CpG island	0.6201
4365	41468_at	Cluster Incl. M30894:Human T-cell receptor Ti rearranged gamma-chain mRNA V-J-C region X14885 /FEATURE=mRNA /DEFINITION=HSTGF31	0.6193
10956	1767_s_at	H.sapiens gene for transforming growth factor-beta 3 (TGF- beta 3)	0.6160
9172	38406_f_at	Cluster Incl. AI207842:ao89h09.x1 Homo sapiens cDNA, 3 end /	0.6155

**Table 3:** Colon cancer: the coefficient of variation (CV) for the original dataset and transformed dataset. This table shows ten features with lowest coefficient of variation, the complete table can be downloaded at .

**Colon Cancer**				**Colon Cancer (Transformed)**		
Rank	No. on array	Gene Acession Name	Coefficient of variation	Rank	Gene Acession Number	Coefficient of variation

1	#39	T57619	45.33%	1	#119/#54	16.53%
2	#119	T51529	48.23%	2	#54/#119	17.19%
3	#54	T48804	48.61%	3	#39/#31	18.88%
4	#58	T71025	49.03%	4	#119/#31	19.85%
5	#365	Z50753	49.60%	5	#31/#39	19.86%
6	#26	T95018	49.79%	6	#31/#119	20.01%
7	#387	U30825	50.74%	7	#39/#119	20.64%
8	#64	H55758	52.48%	8	#119/#39	20.71%
9	#1760	H08393	52.92%	9	#54/#39	20.83%
10	#31	T61609	53.15%	10	#26/#119	21.50%

**Table 4:** Prostate cancer: the coefficient of variation (CV) for the original dataset and transformed dataset according to their rank. This table shows 20 data with lowest coefficient of variation, the complete table can be downloaded at .

**Prostate Cancer**				**Prostate Cancer (transformed)**		
Rank	No. on array	Gene Accession Name	Coefficient of variation	Rank	Gene Acession Number	Coefficient of variation

1	#5871	36845_at	24.54%	1	(#10614)/(#5871)	25.78%
2	#8965	37720_at	25.02%	2	(#7532)/(#6236)	26.75%
3	#8851	37367_at	26.75%	3	(#5871)/(#10614)	27.15%
4	#10614	33198_at	28.47%	4	(#5871)/(#10138)	27.52%
5	#8160	34877_at	28.98%	5	(#9599)/(#10138)	27.94%
6	#5840	36814_at	31.02%	6	(#7715)/(#8889)	27.98%
7	#5954	36928_at	31.77%	7	(#7532)/(#9288)	28.33%
8	#10138	41288_at	31.97%	8	(#8160)/(#10614)	28.41%
9	#6865	39755_at	32.00%	9	(#9424)/(#9599)	28.86%
10	#9599	39551_at	32.07%	10	(#7520)/(#10138)	29.14%

**Table 5:** Colon and prostate cancer: Ten key pair-wise gene expression ratios that are most correlated to tissue phenotype, the complete table can be downloaded at . They were determined to be accurate discriminating axes.

Colon Cancer		Prostate Cancer	
Gene Number	Correlation	Gene Number	Correlation
#481/#67	0.7866	#4751/#6185	0.7454
#1831/#1537	0.7662	#9288/#6185	0.7393
#481/#269	0.7632	#8892/#6185	0.7383
#255/#1760	0.7545	#6185/#8851	0.7371
#481/#508	0.7534	#7532/#6185	0.7349
#481/#768	0.7495	#8136/#6185	0.7335
#1831/#1244	0.7482	#205/#5954	0.7291
#237/#1760	0.7468	#9059/#6185	0.7241
#1482/#1537	0.7460	#4432/#6185	0.7236
#481/#613	0.7369	#8965/#10614	0.721

**Table 6:** Colon cancer: the top 10 genes and pair-wise gene expression ratio used to discriminate the colon cancer tissue. This table is ranked with decreasing classification efficiency. The threshold values *T_i _*for normal tissues are also provided together with classification efficiency. The list of top 25 genes can be downloaded from .

**Colon Cancer-Original data**							**Colon Cancer-Transformed data**				
Rank	No. on array	gene accession number	gene info	Threshold for normal tissue type, *T_j_*	discriminating efficiency*/%	Ref	Rank	gene number on array	Threshold for normal tissue type, *T_i_*	discriminating efficiency*/%	Ref
1	#1659	M26383	Human monocyte-derived neutrophil-activating protein (MONAP) mRNA, complete cds.	<62.7375	87.1%	[12,13]	1	#1537/#1831	<0.75512	93.6%	[27,28]
2	#753	M76378	Human cysteine-rich protein (CRP) gene, exons 5 and 6.	>749.4075	83.9%	[26]	2	#1831/#1537	>1.3243	93.6%	[27,28]
3	#613	X12671	Human gene for heterogeneous nuclear ribonucleoprotein (hnRNP) core protein A1.	<233.4162	82.3%	[33]	3	#1827/#481	<0.074449	91.9%	[14,15,39]
4	#569	T51571	P24480 CALGIZZARIN. SERINE/THREONINE-PROTEIN	<309.3037	77.4%	[34]	4	#1537/#1623	<1.0533	91.9%	[27,40]
5	#1103	R97912	KINASE IPL1 (Saccharomyces cerevisiae)	<70.2738	75.8%	[35]	5	#1831/#1759	>1.4003	91.9%	[28]
6	#1759	J05032	Human aspartyl-tRNA synthetase alpha-2 subunit mRNA, complete cds.	<41.92	75.8%	[36]	6	#1623/#1537	>0.94939	91.9%	[27,40]
7	#241	M63391	Human desmin gene, complete cds.	>2787.0425	75.8%	[17]	7	#365/#1760	>3.3867	91.9%	[41,42]
8	#818	R75843	TRANSLATIONAL INITIATION FACTOR 2 GAMMA SUBUNIT (Homo sapiens)	<152.5662	74.2%	[37]	8	#1759/#1831	<0.71414	91.9%	[28]
9	#1960	T57468	FIBRILLARIN (HUMAN).	<42.0225	74.2%	[38]	9	#1760/#365	<0.29528	91.9%	
10	#1281	H23544	GTP-BINDING NUCLEAR PROTEIN RNA (Homo sapiens)	<103.2488	74.2%	[20]	10	#481/#1827	>13.432	91.9%	[14,15,39]

*discriminating efficiency using only single gene as discriminating axis

**Table 7:** Prostate cancer: the top 10 genes and pair-wise gene expression ratio used to discriminate the prostate cancer tissues. The threshold values *T_i _*for normal tissues are also provided. The list of top 25 genes can be downloaded from .

**Prostate Cancer-Original data**							**Prostate Cancer-Transformed data**				
Rank	No. on array	Probe no	gene info	Threshold Ri	discriminating efficiency* / %	Ref	Rank	gene number on array	Threshold Ri	discriminating efficiency* / %	Ref
1	6185	37639_at	Cluster Incl. X07732:Human hepatoma mRNA for serine protease hepsin	<115	86.3%	[18]	1	#5840/#6185	>0.37168	84.6%	[18,3]
2	10537	33121_g_at	Cluster Incl. AF045229:Homo sapiens regulator of G protein signaling 10 mRNA	<50	80.4%	[43]	2	#6185/#5840	<2.6905	84.6%	[18,31]
3	8965	37720_at	Cluster Incl. M22382:Human mitochondrial matrix protein P1 (nuclear encoded) mRNA	<238	80.4%	[44]	3	#7775/#205	<0.22928	82.7%	[50,51]
4	8554	36589_at	Cluster Incl. X15414:Human mRNA for aldose reductase (EC 1.1.1.2)	>35	79.4%	[45]	4	#8631/#10234	<5.4561	82.7%	[52,53]
5	9172	38406_f_at	Cluster Incl. AI207842:ao89h09.x1 Homo sapiens cDNA	>626	79.4%	[46]	5	#10749/#11942	<0.34585	82.7%	[54]
6	7067	40436_g_at	Cluster Incl. J03592:Human ADP/ATP translocase mRNA	<234	78.4%	[47]	6	#10234/#8631	>0.18328	82.7%	[52,53]
7	9850	40282_s_at	Cluster Incl. M84526:Human adipsin/complement factor D mRNA	>182	77.5%	[30]	7	#8554/#6185	>0.39823	82.7%	[18]
8	7066	40435_at	Cluster Incl. J03592:Human ADP/ATP translocase mRNA, 3 end, clone pHAT8 M96233 /FEATURE=expanded_cds/DEFINITION=HUMGSTM4A Human	<349	76.5%	[47]	8	#11942/#10749	>2.8914	82.7%	[54,55]
9	12153	556_s_at	glutathione transferase class mu number 4(GSTM4) gene	>152	76.5%	[48]	9	#205/#7775	>4.3614	82.7%	[50,51]
10	9093	38087_s_at	Cluster Incl. W72186:zd69b10.s1 Homo sapiens cDNA	>62	74.5%	[49]	10	#6185/#8554	<2.5111	82.7%	[18]

*discriminating efficiency using only single gene as discriminating axis

**Table 8:** Accuracy of N-feature model classifier. The optimum classification accuracy, the mean classification accuracy and the standard deviation for the N-feature classifier (N<11).

**Colon cancer–Original expression data**				**Colon cancer–Transformed expression data**			
**Order or classifier**	Optimum Accuracy* / %	Mean Accuracy* / %	**Standard Deviation**	**Order or classifier**	Optimum Accuracy* / %	Mean Accuracy* / %	**Standard Deviation**

1	87.10%	76.77%	4.17%	1	93.55%	91.24%	1.22%
2	91.94%	83.33%	4.38%	2	98.39%	95.00%	1.95%
3	95.16%	87.07%	4.06%	3	98.39%	96.47%	1.53%
4	95.16%	89.37%	3.58%	4	98.39%	97.20%	1.23%
5	95.16%	90.88%	3.10%	5	98.39%	97.60%	0.99%
6	95.16%	91.94%	2.70%	6	98.39%	97.84%	0.83%
7	95.16%	92.72%	2.38%	7	98.39%	98.00%	0.70%
8	95.16%	93.31%	2.09%	8	98.39%	98.12%	0.60%
9	95.16%	93.78%	1.83%	9	98.39%	98.21%	0.50%
10	95.16%	94.15%	1.57%	10	98.39%	98.28%	0.40%
**Prostate cancer–Original expression data**				**Prostate cancer–Transformed expression data**			

**Order or classifier**	Optimum Accuracy* / %	Mean Accuracy* / %	**Standard Deviation**	**Order or classifier**	Optimum Accuracy* / %	Mean Accuracy* / %	**Standard Deviation**

1	86.27%	75.82%	4.31%	1	84.62%	81.92%	2.28%
2	100.00%	91.27%	7.89%	2	98.39%	90.84%	4.18%
3	100.00%	95.98%	5.53%	3	100.00%	93.64%	3.40%
4	100.00%	97.91%	3.87%	4	100.00%	95.00%	2.94%
5	100.00%	98.86%	2.76%	5	100.00%	95.90%	2.68%
6	100.00%	99.38%	1.98%	6	100.00%	96.59%	2.51%
7	100.00%	99.67%	1.41%	7	100.00%	97.16%	2.36%
8	100.00%	99.83%	0.99%	8	100.00%	97.66%	2.23%
9	100.00%	99.90%	0.67%	9	100.00%	98.10%	2.09%
10	100.00%	99.96%	0.43%	10	100.00%	98.49%	1.94%

**Table 9:** The discriminating axes. The discriminating axes that accounted for the optimum accuracy in 1 to 3-feature model classifier.

**Order or classifier**	Optimum Accuracy* / %	**Discriminating axes**	**Order or classifier**	Optimum Accuracy* / %	**Discriminating axes**
1	87.10%	#1659	1	93.55%	#1831/#1537
2	91.94%	(#241)&(#1659)	2	98.39%	(#753/#768) & (#1831/#1537)
3	95.16%	(#241)&(#1659)&(#1759)	3	98.39%	(#753/#768) & (#1831/#1537)&(#481/#1394)
**Prostate cancer–Original expression data**			**Prostate cancer–Transformed expression data**		

**Order or classifier**	Optimum Accuracy* / %	**Discriminating axes**	**Order or classifier**	Optimum Accuracy* / %	**Discriminating axes**

1	86.27%	(#6185)	1	84.62%	(#6185/#5840)
2	100.00%	(#6185)&(#9850)	2	100.00%	(#6185/#5840)&(#6185/#6749)
3	100.00%	(#6185)&(#9850)&(#12148)	3	100.00%	(#6185/#5840)&(#6185/#6749)&(#7247/#7067)

* : best accuracy based on the specified number of gene/gene ratio as discriminating axes ****Please do not delete from here on, needed for the correct order of reference list****** [32-54]
